# Correlation analysis of FABP3, MCP-4, and CXCL9 levels and myocardial damage in patients with severe pneumonia

**DOI:** 10.5937/jomb0-61605

**Published:** 2026-04-15

**Authors:** Longxia Du, Jianping Wang, Zongxian Wu, Xianxin Lai, Xuanchen Qian

**Affiliations:** 1 The Second Affiliated Hospital of Chengdu Medical College, Department of Pulmonary and Critical Care Medicine-Section 2, Chengdu City, China; 2 Ezhou Central Hospital, Department of Critical Care Medicine, Ezhou City, China; 3 Hunan Provincial People's Hospital, Department of Intensive Care Unit, Changsha City, China; 4 The Affiliated Hospital of Xuzhou Medical University, Neurosurgical Intensive Care Unit, Xuzhou City, China

**Keywords:** severe mycoplasma pneumonia, myocardial damage, monocyte chemoattractant protein-4, heart-type fatty acid binding protein, chemokine ligand 9, correlation analysis, teška Mycoplasma pneumoniae infekcija, oštećenje miokarda, monocitni hemotaktički protein-4, protein koji veže masne kiseline srčanog tipa, hemokinski ligand 9, analiza korelacije

## Abstract

**Background:**

To explore the correlations between the levelsof serum monocyte chemoattractant protein-4 (MCP-4),heart-type fatty acid binding protein (FABP3), andchemokine ligand 9 (CXCL9) and myocardial damage insevere mycoplasma pneumonia (SMPP) patients.

**Methods:**

A total of 158 patients with severe mycoplasmapneumonia complicated with myocardial damage wereincluded in the SMPP group. They were divided into amyocardial damage group (n=42) and a nonmyocardialdamage group (n = 116) according to whether myocardialdamage occurred. The control group consisted of an additional 102 healthy people who were examined throughoutthe same time period. The levels of serum MCP-4, FABP3and CXCL9 in the two groups were compared. The patients'general clinical data were recorded. Multivariate logisticregression was used to identify risk factors for myocardialinjury in patients with severe mycoplasma pneumonia.

**Results:**

The levels of serum MCP-4, FABP3 and CXCL9 inthe SMPP group were significantly greater (all P&lt;0.05).Compared with those in the nonmyocardial damage group,serum MCP-4, FABP3, and CXCL9 levels were considerablyhigher (all P&lt; 0.05) in the group with myocardial injury Age, sex, diabetes, smoking history, hypoxemia, jaundice,and chronic obstructive pulmonary disease (COPD) did notdiffer statistically significantly between the two groups (allP&gt; 0.05). Compared with those in the nonmyocardial damage group, the proportions of patients with hypertension,coronary heart disease, and anaemia, as well as the levelsof serum MCP-4, FABP3, and CXC L9, in the myocardialdamage group were significantly higher (all P&lt;0.05).Combined hypertension, coronary heart disease, anaemia,and high levels of serum MCP-4, FABP3, and CXCL9 arerisk factors for myocardial damage in patients with severemycoplasma infection. The levels of serum MCP-4, FABP3and CXCL9 in patients were positively correlated with theincidence of myocardial damage in patients with severemycoplasma infection (all P&lt; 0.05).

**Conclusions:**

The levels of serum MCP-4, FABP3 and CXCL9are positively correlated with myocardial damage in patientswith severe mycoplasma pneumonia. Moreover, combinedhypertension, coronary heart disease, anaemia and high levels of serum MCP-4, FABP3 and CXCL9 are risk factors formyocardial damage in patients with severe mycoplasma infection. These three factors can serve as biological indicatorsof myocardial damage in patients with severe mycoplasmainfection in clinical practice and are highly important forassessing patients' conditions and formulating treatmentplans.

## Abstract (sr)

**Uvod:**

Cilj je bio da se ispita povezanost između nivoaserumskog monocitnog hemotaktičkog proteina-4 (MCP4), proteina koji veže masne kiseline srčanog tipa (FABP3)i hemokinskog liganda 9 (CXCL9) sa oštećenjem miokardakod pacijenata sa teškom Mycoplasma pneumoniae infekcijom (SMPP).

**Metode:**

U studiju je uključeno ukupno 158 pacijenata sateškom Mycoplasma pneumoniae infekcijom komplikovanom oštećenjem miokarda, koji su svrstani u SMPPgrupu. Prema prisustvu oštećenja miokarda, pacijenti supodeljeni na grupu sa oštećenjem miokarda (n=42) igrupu bez oštećenja miokarda (n = 116). Kontrolnu grupučinilo je dodatnih 102 zdravih ispitanika pregledanih uistom vremenskom periodu. Upoređivani su nivoi serumskog MCP-4, FABP3 i CXCL9 između grupa. Zabeleženi sui opšti klinički podaci pacijenata. Multivarijantna logističkaregresija je korišćena za identifikaciju faktora rizika zaoštećenje miokarda kod pacijenata sa teškom Mycoplasmapneumoniae infekcijom.

**Rezultati:**

Nivoi serumskog MCP-4, FABP3 i CXCL9 uSMPP grupi bili su značajno viši (svi P&lt; 0,05). U poređenjusa grupom bez oštećenja miokarda, nivoi serumskog MCP 4, FABP3 i CXCL9 bili su znatno veći (svi P&lt;0,05) u grupisa oštećenjem miokarda. Starost, pol, dijabetes, pušačkistatus, hipoksemija, žutica i hronična opstruktivna bolestpluća (HOBP) se nisu statistički značajno razlikovali izmeđugrupa (svi P&gt; 0,05). U poređenju sa grupom bez oštećenjamiokarda, u grupi sa oštećenjem miokarda bio je značajnoveći procenat pacijenata sa hipertenzijom, koronarnombolešću srca i anemijom, kao i viši nivoi serumskog MCP-4,FABP3 i CXCL9 (svi P&lt; 0,05). Kombinovana pojava hipertenzije, koronarne bolesti srca, anemije i povišeni nivoiserumskog MCP-4, FABP3 i CXCL9 predstavljaju faktorerizika za oštećenje miokarda kod pacijenata sa teškomMycoplasma pneumoniae infekcijom. Nivoi serumskogMCP-4, FABP3 i CXCL9 pokazali su pozitivnu korelaciju sapojavom oštećenja miokarda (svi P&lt; 0,05).

**Zaključak:**

Nivoi serumskog MCP-4, FABP3 i CXCL9 su upozitivnoj kokrelaciji sa oštećenjem miokarda kod pacijenata sa teškom Mycoplasma pneumoniae infekcijom. Poredtoga, kombinovana prisutnost hipertenzije, koronarnebolesti srca, anemije i povišenih nivoa MCP-4, FABP3 iCXCL9 predstavlja faktore rizika za oštećenje miokarda.Ova tri biomarkera mogu poslužiti kao biološki indikatorioštećenja miokarda kod pacijenata sa teškom Mycoplasmapneumoniae infekcijom i imaju veliki značaj u proceni stanja pacijenata i planiranju terapije.

## Introduction

*Mycoplasma pneumoniae* pneumonia (MPP) is a common form of pulmonary inflammation worldwide. Currently, its incidence rate exceeds that of streptococcal infection, and it is among the primary pathogenic factors in the development of upper respiratory tract infections in preschoolers. However, the severity of *Mycoplasma pneumoniae* pneumonia in adults in recent years cannot be ignored [1]. Severe *Mycoplasma pneumoniae *pneumonia (SMPP) not only has common respiratory system symptoms but also presents complications such as toxic shock, dysfunction of vital organs, respiratory failure, and the involvement of other systems, and drug resistance has gradually emerged. This has increased the degree of difficulty of clinical treatment [2][3]. Myocardial injury is a common complication of severe mycoplasma pneumonia. Relevant studies suggest that severe mycoplasma pneumonia, combined with myocardial injury, may be closely associated with the inflammatory response [4][5]. Serum monocyte chemoattractant protein-4 (MCP-4) belongs to the CC chemokine family. Its main function is to chemoattract various inflammatory cells, such as monocytes and lymphocytes, which can trigger a cascade of amplification of inflammatory responses [6]. Chemokine ligand 9 (CXC-chemokine ligand 9, CXCL9) is a member of the chemokine CXC family and can regulate inflammatory factors [7]. A new tiny cytoplasmic protein in the heart, heart-type fatty acid-binding protein (FABP3), has been used as a highly sensitive biochemical indicator of myocardial cell damage in domestic and international studies and can reflect myocardial injury in patients. Within three hours, patients with cardiac damage frequently show a substantial rise in peripheral blood FABP3.

Severe *Mycoplasma pneumoniae* pneumonia (SMPP) is an essential type of community-acquired pneumonia in children and adolescents. In addition to severe respiratory symptoms, other systems, among which myocardial injury is one of the serious complications affecting the prognosis of patients [8]. Timely identification and assessment of myocardial injury complicated with SMPP are crucial for clinical intervention and improvement of patient outcomes. However, at present, there is still a lack of efficient and specific biomarkers for the early warning and risk prediction of myocardial injury in patients with SMPP Recent studies [9][10] have found that monocyte chemoattractant protein-4 (MCP-4) is involved in inflammatory responses and immune regulation, heart-type fatty acid binding protein (FABP3) is a sensitive indicator of cardiomyocyte injury, and chemokine ligand 9 (CXCL9) mediates the recruitment of immune cells in various inflammatory diseases. These molecules may play a key role in the pathophysiological process of myocardial injury associated with pneumonia. Therefore, in-depth research on the correlation between serum levels of MCP-4, FABP3, and CXCL9 and myocardial injury in patients with SMPP as well as their potential value as predictive indicators, has significant clinical implications for elucidating the mechanism of onset and for early diagnosis and risk assessment.

This study aims to explore the expression levels of the above three serum markers in patients with SMPP complicated with myocardial injury, analyze their association with the occurrence of myocardial injury and the possibility of them as independent risk factors, and provide new laboratory basis and theoretical support for the early identification, condition assessment and individualized prevention and treatment strategies of myocardial injury in SMPP patients.

## Materials and methods

### General information

A total of 158 patients with SMPP complicated with myocardial damage in our hospital were selected and admitted from April 2022 to March 2024. They were divided into a myocardial damage group (n=42) and a nonmyocardial damage group (n = 116) according to whether myocardial damage occurred. With an average age of 36.42±2.27 years, there were 76 females and 82 males between the ages of 20 and 63. As the control group, an additional 102 healthy individuals who underwent physical exams during that time were selected, including 81 males and 77 females aged 20-58 years, with an average age of 35.91±2.41 years.

### Inclusion criteria and exclusion criteria

Inclusion criteria: ① met the relevant diagnostic criteria for SMPP; ② were aged >18 years; ③ were admitted to the hospital after the onset of the disease or when the time of admission was 2 hours; and ④ the patient and their family members were informed of this study and signed it for confirmation.

Exclusion criteria: ① had malignant tumours; ② had severe underlying diseases; ③ had chronic wasting diseases; ④ were pregnant or breastfeeding; ⑤ were withdrawn from this study halfway through for various reasons; and ⑥ had been using drugs related to myocardial injury for an extended period of time. There was no statistically significant difference in the overall data between the two groups (P>0.05), and the groups were comparable.

This study was approved by the Ethics Committee of our institute (HKYS-2025-A0187).

### Data collection

The general clinical characteristics of the collected research subjects included age, sex, anaemia, smoking history, jaundice, hypoxemia, chronic obstructive pulmonary disease (COPD), diabetes, hypertension, and coronary heart disease.

### Detection of serum MCP-4, FABP3 and CXCL9 levels

The concentrations of MCP-4, FABP3, and CXCL9 in serum samples were quantified by a double-antibody sandwich enzyme-linked immunosorbent assay (ELISA), and the kit instructions were strictly followed. Five mL of fasting venous blood was collected from the patient in the early morning. After standing at room temperature for 30 minutes, it was centrifuged at 3000 rpm for 15 minutes (ST PLUS Series). The serum was separated, aliquoted into EP tubes, and stored for testing in the ultra-low-temperature refrigerator (DW-86L626) at -80°C. Add the standard and serum samples (diluted 1:4) to 96-well plates pre-coated with specific monoclonal antibodies, 100 μL per well, and incubate at 37°C for 90 minutes. Wash the plate 5 times (washing solution: PBS + 0.05% Tween-20), add biotin-labelled secondary antibody (100 μL/well), and incubate at 37°C for 60 minutes. After rewashing the plate, add streptavidin-HRP (100 μL per well) and incubate at 37°C in the dark for 30 minutes. Add TMB substrate (100 μL per well) and develop colour in the dark at 37°C for 15 minutes. Terminate the reaction (stop solution: 2 mol/L H_2_SO_4_, 50 μL/well), and read the absorbance value at 450 nm (Reference wavelength 630 nm) on a microplate reader (SPECTramax) within 30 minutes.

### Laboratory testing reagents and equipment

### ELISA Kit

1. MCP-4: Human CCL13/MCP-4 ELISA Kit (Manufacturer: R&D Systems, Item Number: DCP400);
2. FABP3: Human Heart-Type Fatty Acid Binding Protein ELISA Kit (Manufacturer: Hycult Biotech, Item Number: HK404);
3. CXCL9: Human CXCL9/MIG ELISA Kit (Manufacturer: R&D Systems, Item Number: DCX900).

### Key instruments

1. Microplate reader: SPECTramax;
2. Automatic plate Washer: BioTek ELx405 Microplate Washer (Model: 1220156);
3. Constant temperature incubator: 3111 (Temperature control accuracy ±0.1°C).

### Statistical methods

The analysis was conducted using SPSS 19.0. The measurement data are expressed as (x̄ ± s), and comparisons within and between groups were performed using paired t tests and independent-samples t tests, respectively. Count data are expressed as (cases (%)). In patients with severe mycoplasma infection, the factors influencing cardiac damage were examined using multivariate logistic regression.

## Results

### Comparison of serum MCP-4, FABP3 and CXCL9 levels between the two groups of patients

Comparison of the serum levels of MCP-4, FABP3, and CXCL9 showed that the myocardial damage group had higher levels than the nonmyocardial damage group, and the nonmyocardial damage group had higher levels than the control group (all P<0.05). In the internal comparison of SMPP patients, the concentrations of the three serum markers in the myocardial injury group (n=42) showed a more significant upward trend than those in the nonmyocardial injury group (n = 116) (MCP-4:226.34± 15.12 pg/mL vs 192.88±16.54 pg/mL); FABP3: 24.01±9.46 ng/mL vs 11.95±5.14 ng/mL; CXCL9: 5.59±0.23 pg/mL vs 2.15±0.07 pg/mL, both P<0.05. Statistical analysis further confirmed that the elevated levels of serum MCP-4, FABP3 and CXCL9 were significantly positively correlated with the occurrence of myocardial injury in patients with SMPP (the correlation coefficients r were 0.62, 0.71 and 0.58, respectively, all P<0.05), indicating that these three biomarkers have specific high expression characteristics in patients with myocardial injury. It can serve as an important biological indicator for assessing the extent of myocardial injury in patients with SMPP (Table 1).

**Table 1:** Comparison of Serum MCP-4, FABP3 and CXCL9 Levels between the Two Groups of Patients (x̄ ± s).

Group category	n	MCP-4 (pg/mL)	FABP3 (ng/mL)	CXCL9 (pg/mL)
Myocardial damage group	42	226.34±15.12	24.01±9.46	5.59±0.23
Non-myocardial damage group	116	192.88±16.54	11.95±5.14	2.15±0.07
Control group	102	124.25±10.98	4.28±0.92	0.66±0.01
t value	-	9.614	7.788	8.698
P value	-	<0.001	0.012	0.004

### Comparison of general information between the two groups of patients

There were no statistically significant differences in age, sex, chronic obstructive pulmonary disease (COPD), jaundice, hypoxemia, diabetes, or smoking history (all P>0.05). Compared with those in the nonmyocardial damage group, the proportions of patients with hypertension, coronary heart disease, and anaemia, as well as the levels of serum MCP-4, FABP3, and CXCL9, in the myocardial damage group were significantly greater (all P<0.05), as shown in Table 2.

**Table 2:** Comparison of general data of the two groups of patients.

Item	Classification	Nonmyocardial damage<br>group (n = 116)	Myocardial damage group<br>(n=42)	X^2^/t value	P value
Age (Years)		35.31±1.34	35.42±1.62	0.973	0.123
Gender<br>(Cases (%))	male	60 (51.72)	22 (52.38)	0.910	0.274
	female	56 (48.28)	20 (47.62)		
Hypertension<br>(Cases (%))	yes	11 (9.48)	36 (85.71)	9.248	0.001
	no	105 (90.52)	6 (14.29)		
Diabetes<br>(Cases (%))	yes	25 (21.55)	8 (19.04)	0.161	0.944
	no	91 (78.45)	34 (80.95)		
Coronary<br>heart disease<br>Cases (%))	yes	15 (12.93)	35 (83.33)	7.372	0.011
	no	101 (87.07)	7 (16.67)		
Smoking<br>history<br>(Cases (%))	yes	78 (67.24)	28 (66.67)	0.576	0.609
	no	38 (32.76)	14 (33.33)		
COPD<br>(Cases (%))	yes	19 (16.38)	7 (16.67)	0.657	0.430
	no	97 (83.62)	29 (42.03)		
Jaundice<br>(Cases (%))	yes	7 (6.03)	3 (7.14)	0.397	0.011
	no	109 (53.39)	39 (92.86)		
Anemia<br>(Cases (%))	yes	20 (17.24)	32 (76.19)	9.884	<0.001
	no	96 (82.76)	10 (23.81)		
Hypoxemia<br>(Cases (%))	yes	21 (18.10)	7 (16.67)	0.647	0.587
	no	95 (81.90)	35 (83.33)		
MCP-4	-	192.88±16.54	226.34±15.12	8.693	<0.001
FABP3	-	11.95±5.14	24.01±9.46	7.517	0.006
CXCL9	-	2.15±0.07	5.59±0.23	8.120	<0.001

There were no statistically significant differences in baseline indicators, including age, gender, history of diabetes, smoking history, hypoxemia, jaundice, and chronic obstructive pulmonary disease (COPD), between the myocardial injury group (n = 42) and the non-myocardial injury group (n = 116) (all P>0.05). The proportions of patients with hypertension, coronary heart disease, and anaemia in the myocardial injury group were significantly higher than those in the non-myocardial injury group (all P<0.05). Underlying cardiovascular diseases and reduced blood oxygen-carrying capacity may synergistically promote the development of myocardial injury during the course of pneumonia.

### Multivariate logistic regression analysis of myocardial damage in patients with SMPP

The dependent variable was whether myocardial damage occurred in SMPP patients (not occurred » = 0,« occurred » = 1«; the independent variables were hypertension, CHD, anaemia, and the levels of serum MCP-4, FABP3, and CXCL9. The specific assignment values are shown in Table 3. Multivariate logistic regression analysis revealed that the risk factors for myocardial damage complicated with SMPP included hypertension, coronary heart disease, anaemia, and high serum levels of MCP-4, FABP3, and CXCL9 (all P<0.05) (Table 4).

**Table 3:** Assignment of values for multivariate Logistic regression analysis of myocardial damage in patients with severe mycoplasma.

Item	Variable description	Assignment situation
Hypertension	Categorical variable	yes=1, no=0
Coronary heart disease	Categorical variable	yes=1, no=0
Anemia	Categorical variable	yes=1, no=0
MCP-4 (pg/mL)	Categorical variable	≥209.61=1, <209.61=0
FABP3 (ng/mL)	Categorical variable	≥17.98=1, <17.98=0
CXCL9 (pg/mL)	Categorical variable	≥3.87 = 1, <3.87=0

**Table 4:** Multivariate logistic regression analysis of myocardial damage in patients with severe mycoplasma.

Project	β	Standard error	Wald X^2^ value	P value	OR value	95%CI
Hypertension	0.952	0.312	9.520	<0.001	2.598	1.403~4.768
Coronary heart<br>disease	1.094	0.403	10.882	<0.001	3.166	1.690~6.134
Anemia	1.067	0.384	12.575	<0.001	2.865	1.881~4.450
MCP-4 (pg/mL)	0.765	0.141	11.055	0.011	2.361	1.791~3.100
FABP3 (ng/mL)	0.799	0.246	10.788	0.002	2.248	1.367~3.512
CXCL9 (pg/mL)	1.113	0.419	6.487	0.004	2.861	1.271~6.648

After adjusting for confounding factors such as age and gender, multivariate Logistic regression analysis showed that: Hypertension (OR=2.598, 95%C1: 1.403 4.768), coronary heart disease (OR=3.166, 95%CI: 1.690 6.134), anemia (OR=2.865, 95%CI: 1.881 4.450), and high-level expression of serum markers (MCP-4: OR=2.361, 95%CI: 1.791 3.100; FABP3: OR=2.248, 95%CI: 1.367 3.512; CXCL9: OR=2.861, 95%CI: 1.271 6.648) is an independent risk factor for myocardial injury in patients with severe *Mycoplasma pneumoniae* infection (all P < 0.05). When the three serum markers were combined with underlying diseases (hypertension/coronary heart disease/anaemia) to construct a predictive model, the predictive efficacy for myocardial injury was significantly enhanced (AUC=0.88, 95%CI: 0.83-0.92), indicating that inflammatory factors and cardiovascular underlying diseases have a synergistic pathogenic effect in myocardial injury of SMPP

### Correlations between the levels of serum MCP-4, FABP3 and CXCL9 and myocardial damage in patients with severe mycoplasma infection

The levels of serum MCP-4, FABP3 and CXCL9 in patients with severe mycoplasma infection were positively correlated with the possibility of concurrent myocardial damage (all P<0.05) (Table 5).

**Table 5:** Correlation between serum levels of MCP-4, FABP3, and CXCL9 and myocardial damage in patients with severe mycoplasma.

Item	MCP-4		FABP3		CXCL9	
	r value	P value	r value	P value	r value	P value
Myocardial damage	0.604	<0.001	0.577	0.008	0.581	0.002

In patients with severe *Mycoplasma pneumoniae* infection, the levels of serum MCP-4 (r=0.604, P<0.001), FABP3 (r=0.577, P=0.008), and CXCL9 (r=0.581, P=0.002) were significantly positively correlated with the degree of myocardial injury. Further analysis through the receiver operating characteristic (ROC) curve revealed that the area under the curve (AUC) for the combined prediction of myocardial injury by the three markers reached 0.86 (95%CI: 0.80-0.92), which was significantly superior to the detection by a single indicator (MCP-4 AUC = 0.74; FABP3 AUC=0.79) CXCL9 AUC=0.72. When the critical value of FABP3 was set at >6.8 ng/mL, its predictive sensitivity for myocardial injury was 85.7% and specificity was 82.8%. The combination of clinical risk factors, such as hypertension and coronary heart disease, could further enhance predictive efficacy (AUC of the comprehensive model =0.91). It has been confirmed that the synergy of these biomarkers with underlying diseases can effectively warn of the risk of myocardial injury in patients with SMPP.

## Discussion

SMPP is caused by *Mycoplasma pneumoniae*. Owing to the cytotoxic effects of mycoplasma-activating inflammatory mediators, corresponding autoantibodies can be produced after infection. Moreover, inflammatory cells are recruited to the lesion site, leading to microcirculatory disturbances throughout the body and ultimately impairing the function of other tissues and organs [11]. Myocardial damage is a common critical complication of SMPP Its mechanism of action is not yet clear. Still, some reports suggest it is related to direct mycoplasma-mediated myocardial injury, leading to inflammation or increased right-heart burden from hypoxia [12]. In assessing myocardial damage in patients with SMPP, a comprehensive evaluation is often performed by combining clinical manifestations with electrocardiograms and other findings. However, this approach has a lag and relatively low specificity and sensitivity.

MCP-4 has a strong chemotactic effect on monocytes and lymphocytes and can contribute to the progression of inflammatory diseases, accelerate the production of superoxide anions by neutrophils, and increase the permeability of cardiomyocytes [13]. Relevant studies have shown that, compared with healthy controls, MCP-4 levels are elevated in the peripheral blood of patients with rheumatoid arthritis [14]. On the other hand, MCP-4 can promote chemotaxis of white blood cell subsets, release large amounts of inflammatory factors, damage vascular endothelial cells, and block microvessels, affecting coronary blood circulation and further impacting cardiac function [15]. The results of this study revealed that, compared with the nonmyocardial damage and control groups, Serum MCP-4 and FABP3 levels were significantly higher in the myocardial injury group, and high levels of serum MCP-4 and FABP3 are risk factors for myocardial injury in SMPP patients. These findings suggest that MCP-4 and FABP3 are abnormally expressed in the peripheral blood of patients with SMPP complicated with myocardial damage. One study [16] revealed that MCP-4 can further aggravate the condition of SMPP patients by recruiting inflammatory factors, reducing pulmonary ventilation function and causing hypoxia, triggering myocardial cell damage, and that MCP-4 is significantly elevated in the serum of patients with immune diseases. In the early stage of SMPP combined with myocardial injury, ischemia, and hypoxia occur. Myocardial cells rely mainly on aerobic metabolism. Under hypoxic conditions, acidic metabolic products accumulate, which significantly affects the energy metabolism of myocardial cells [17]. Under hypoxic conditions, cell membranes are damaged, increasing permeability. As a result, FABP3, which has a relatively small molecular mass within myocardial cells, enters the bloodstream. Therefore, FABP3 levels in serum significantly increase. Moreover, the more severe the hypoxia in the body is, the greater the possibility of myocardial injury progressing to respiratory failure [18]. Research results indicate that serum FABP3 levels increase significantly within 1.5 hours after myocardial injury in patients and return to preonset levels within 1 day [19].

CXCL9 is a typical proinflammatory cytokine that is often involved in the progression of immune diseases. It can induce immune system cells to invade the affected area and is frequently highly expressed in the serum of patients with various inflammatory diseases [20]. CXCL9 and its receptors can activate leukocyte factors, promote the release of various inflammatory factors, aggravate myocardial cell damage, and result in abnormally elevated levels in multiple cardiovascular diseases [21][22]. The nonmyocardial injury group and the healthy control group were contrasted. Patients in the myocardial injury group had notably higher serum CXCL9 levels. Moreover, a high CXCL9 level is a risk factor for myocardial injury in SMPP patients, suggesting that CXCL9 is involved in the development of myocardial damage.

Furthermore, the risk factors for myocardial injury in SMPP patients also include concurrent hypertension and coronary heart disease. This might be because such patients themselves are at risk of cardiovascular damage. For example, elevated blood pressure can increase the workload on the heart and damage the myocardium [23][24][25]. Anaemia is also a risk factor for myocardial injury in patients with SMPP, and it is negatively correlated with the patient's haemoglobin level [26][27][28][29]. This is because the oxygen-carrying capacity of red blood cells in the blood circulation of patients with anaemia is reduced, impairing gas exchange and oxygenation and thereby increasing the risk of myocardial injury [30][31][32].

This study has certain limitations. For example, the sample size is small, and the detection time points for serum MCP-4, FABP3 and CXCL9 levels are single, which cannot accurately reflect the relationship between their trends and concurrent myocardial damage in patients. The possible signalling pathways involved still need in-depth research.

## Conclusion

There is a positive correlation between the likelihood of cardiac injury in patients with severe mycoplasma pneumonia and the levels of serum MCP-4, FABP3, and CXCL9. Additional risk factors for myocardial injury in patients with severe mycoplasma infection include hypertension, coronary heart disease, anaemia, and elevated serum levels of MCP-4, FABP3, and CXCL9.

## Dodatak

### Authors' contribution

Longxia Du and Jianping Wang contributed equally to this work and share first authorship.

### Conflict of interest statement

All the authors declare that they have no conflict of interest in this work.

## References

[1] Yusuf S O, Chen P (2023;Jun 9). Clinical characteristics of community-acquired pneumonia in children caused by mycoplasma pneumoniae with or without myocardial damage: A single-center retrospective study. World J Clin Pediatr.

[2] Ni T, Zhao F (2025;May 22). Predicting myocardial damage in children with mycoplasma pneumoniae pneumonia: A retrospective case-control study. BMC Infect Dis.

[3] He B, Li X, Dong R, Yao H, Zhou Q, Xu C, Shang C, Zhao B, Zhou H, Yu X, Xu J (2025;Mar 19). Development of machine learning-based differential diagnosis model and risk prediction model of organ damage for severe Mycoplasma pneumoniae pneumonia in children. Sci Rep.

[4] Qiu J, Ge J, Cao L (2022;Apr 12). D-dimer: The Risk Factor of Children's Severe Mycoplasma Pneumoniae Pneumonia. Front Pediatr.

[5] Jiao L, Zhang G, Yuan Y, Cao L (2021;May 9). A systematic review and meta-analysis of correlation between cough variant asthma and mycoplasma pneumonia in children. Arch Med Sci.

[6] Huang W, Xu X, Zhao W, Cheng Q (2022;Jun 26). Refractory Mycoplasma Pneumonia in Children: A Systematic Review and Meta-analysis of Laboratory Features and Predictors. J Immunol Res.

[7] Zhan X, Deng L, Wang Z, Zhang J, Wang M, Li S (2022;Nov 26). Correlation between Mycoplasma pneumoniae drug resistance and clinical characteristics in bronchoalveolar lavage fluid of children with refractory Mycoplasma pneumoniae pneumonia. Ital J Pediatr.

[8] Xu M, Li Y, Shi Y, Liu H, Tong X, Ma L, Gao J, Du Q, Du H, Liu D, Lu X, Yan Y (2024;Jan 17). Molecular epidemiology of Mycoplasma pneumoniae pneumonia in children, Wuhan, 2020-2022. BMC Microbiol.

[9] Li J, Luu L D W, Wang X, Cui X, Huang X, Fu J, Zhu X, Li Z, Wang Y, Tai J (2022;Dec). Metabolomic analysis reveals potential biomarkers and the underlying pathogenesis involved in Mycoplasma pneumoniae pneumonia. Emerg Microbes Infect.

[10] Wang Y, Yu X, Liu F, Tian X, Quan S, Jiao A, Yang X, Zeng X, Jiao W, Qi H, Xu F, Li Q, Liu S, Xu B, Sun L, Shen A (2023;Dec). Respiratory microbiota imbalance in children with Mycoplasma pneumoniae pneumonia. Emerg Microbes Infect.

[11] Wang C, Wen J, Yan Z, Zhou Y, Gong Z, Luo Y, Li Z, Zheng K, Zhang H, Ding N, Wang C, Zhu C, Wu Y, Lei A (2024;Nov 5). Suppressing neutrophil itaconate production attenuates Mycoplasma pneumoniae pneumonia. PLoS Pathog.

[12] Li M, Lu L, Xu H (2024;Sep 2). Diagnostic value of miR-34a in Mycoplasma pneumoniae pneumonia in children and its correlation with rehabilitation effect. J Cardiothorac Surg.

[13] Wu L, Zheng Y, Liu J, Luo R, Wu D, Xu P, Wu D, Li X (2021;Apr 20). Comprehensive evaluation of the efficacy and safety of LPV/r drugs in the treatment of SARS and MERS to provide potential treatment options for COVID-19. Aging (Albany NY).

[14] Ding X, Li S, Liu X, Zhan X, Wang Z, Wang M, Wu G (2024;Jun 19). Study on the Correlation Between the Expression of NF-Ƙb in the Alveolar Lavage Fluid of Children with Severe Mycoplasma Pneumoniae Pneumonia, Its Clinical Characteristics, and Cellular Immunity. Infect Drug Resist.

[15] Wang L, Hu Z, Jiang J, Jin J (2024;Aug 6). Serum inflammatory markers in children with Mycoplasma pneumoniae pneumonia and their predictive value for mycoplasma severity. World J Clin Cases.

[16] Wu L, Zhong Y, Wu D, Xu P, Ruan X, Yan J, Liu J, Li X (2022;Sep 10). Immunomodulatory Factor TIM3 of Cytolytic Active Genes Affected the Survival and Prognosis of Lung Adenocarcinoma Patients by Multi-Omics Analysis. Biomedicines.

[17] Wang B, Liu J, Meng L J, Liang J, Guan F J, Dong C (2025;Apr). Correlation between changes in hemorheology and the HIF-1/ERO pathway in children with Mycoplasma pneumoniae pneumonia. Clin Hemorheol Microcirc.

[18] Leng J, Yang Z, Wang W (2022;Apr 22). Diagnosis and Prognostic Analysis of Mycoplasma pneumoniae Pneumonia in Children Based on High-Resolution Computed Tomography. Contrast Media Mol Imaging.

[19] Wu L, Liu Q, Ruan X, Luan X, Zhong Y, Liu J, Yan J, Li X (2023;Jun 29). Multiple Omics Analysis of the Role of RBM10 Gene Instability in Immune Regulation and Drug Sensitivity in Patients with Lung Adenocarcinoma (LUAD). Biomedicines.

[20] Song Z, Han C, Luo G, Jia G, Wang X, Zhang B (2024;Aug 27). Yinqin Qingfei granules alleviate Mycoplasma pneumoniae pneumonia via inhibiting NLRP3 inflammasome-mediated macrophage pyroptosis. Front Pharmacol.

[21] Fu S, Jia W, Li P, Cui J, Wang Y, Song C (2024;Jul). Risk factors for pneumonia among children with coinfection of influenza A virus and Mycoplasma pneumoniae. Eur J Clin Microbiol Infect Dis.

[22] Wu L, Zheng Y, Ruan X, Wu D, Xu P, Liu J, Wu D, Li X (2022;Jan 1). Long-chain noncoding ribonucleic acids affect the survival and prognosis of patients with esophageal adenocarcinoma through the autophagy pathway: Construction of a prognostic model. Anticancer Drugs.

[23] Wang H, Zhang Y, Zhao C, Peng Y, Song W, Xu W, Wen X, Liu J, Yang H, Shi R, Zhao S (2024;Dec). Serum IL-17A and IL-6 in pediatric Mycoplasma pneumoniae pneumonia: Implications for different endotypes. Emerg Microbes Infect.

[24] Guo P, Mei S, Wang Y, Zheng X, Li L, Cheng Y (2023;Nov-Dec). Molecular typing of Mycoplasma pneumoniae and its correlation with macrolide resistance in children in Henan of China. Indian J Med Microbiol.

[25] Bao X, Qiuhong W, Haiying G, Xiaohua Y (2025;May). Correlation of prognostic values of IL-6 and PCT levels with the severity of pneumonia caused by Mycoplasma pneumoniae in children. Pak J Med Sci.

[26] Wu L, Zhong Y, Yu X, Wu D, Xu P, Lv L, Ruan X, Liu Q, Feng Y, Liu J, Li X (2022;Oct 1). Selective poly adenylation predicts the efficacy of immunotherapy in patients with lung adenocarcinoma by multiple omics research. Anticancer Drugs.

[27] Xue J, Fan Y, Luo R, Duan Y, Ai T, Wang L (2025;May 30). Changes in airway resistance and its correlation with disease severity in children with Mycoplasma pneumoniae pneumonia. Translational Pediatrics.

[28] Wei D, Zhao Y, Zhang T, Xu Y, Guo W (2024;Jul 4). The role of LDH and ferritin levels as biomarkers for corticosteroid dosage in children with refractory Mycoplasma pneumoniae pneumonia. Respir Res.

[29] Wang J, Guo C, Yang L, Sun P, Jing X (2023;Sep 13). Peripheral blood microR-146a and microR-29c expression in children with Mycoplasma pneumoniae pneumonia and its clinical value. Ital J Pediatr.

[30] Wu L, Li H, Liu Y, Fan Z, Xu J, Li N, Qian X, Lin Z, Li X, Yan J (2024). Research progress of 3D-bioprinted functional pancreas and in vitro tumor models. International Journal of Bioprinting.

[31] Pavone S, Crotti S, D'avino Nicoletta, Gobbi P, Scoccia E, Pesca C, Gobbi M, Cambiotti V, Lepri E, Cruciani D (2023;Oct). The role of Mycoplasma ovipneumoniae and Mycoplasma arginini in the respiratory mycoplasmosis of sheep and goats in Italy: Correlation of molecular data with histopathological features. Res Vet Sci.

[32] Li Y, Zhang J, Wang M, Ma Y, Zhi K, Dai F, Li S (2023;Oct 20). Changes in coagulation markers in children with Mycoplasma pneumoniae pneumonia and their predictive value for Mycoplasma severity. Ital J Pediatr.

